# A Healthy Dietary Pattern Reduces Lung Cancer Risk: A Systematic Review and Meta-Analysis

**DOI:** 10.3390/nu8030134

**Published:** 2016-03-04

**Authors:** Yanlai Sun, Zhenxiang Li, Jianning Li, Zengjun Li, Jianjun Han

**Affiliations:** 1 Department of Surgical Oncology, Shandong Cancer Hospital and Institute, No. 440 Jiyan Road, Jinan 250117, China; sunyanlaisd@163.com (Y.S.); lizjdear@163.com (Z.L.); 2 Department of Radiation Oncology, Shandong Cancer Hospital and Institute, No. 440 Jiyan Road, Jinan 250117, China; lizjj123@163.com; 3 Department of Anesthesiology and Operation, Affiliated Hospital of Shandong Academy of Medical Sciences, 38 Wuyingshan Road, Jinan 250031, China; jianningleee@163.com

**Keywords:** nutrition, dietary pattern, lung cancer, risk factors, meta-analysis

## Abstract

Background: Diet and nutrients play an important role in cancer development and progress; a healthy dietary pattern has been found to be associated with several types of cancer. However, the association between a healthy eating pattern and lung cancer risk is still unclear. Objective: Therefore, we conducted a systematic review with meta-analysis to evaluate whether a healthy eating pattern might reduce lung cancer risk. Methods: We identified relevant studies from the PubMed and Embase databases up to October 2015, and the relative risks were extracted and combined by the fixed-effects model when no substantial heterogeneity was observed; otherwise, the random-effects model was employed. Subgroup and publication bias analyses were also performed. Results: Finally, eight observational studies were included in the meta-analysis. The pooled relative risk of lung cancer for the highest *vs.* lowest category of healthy dietary pattern was 0.81 (95% confidence interval, CI: 0.75–0.86), and no significant heterogeneity was detected. The relative risks (RRs) for non-smokers, former smokers and current smokers were 0.89 (95% CI: 0.63–1.27), 0.74 (95% CI: 0.62–0.89) and 0.86 (95% CI: 0.79–0.93), respectively. The results remained stable in subgroup analyses by other confounders and sensitivity analysis. Conclusions: The results of our meta-analysis suggest that a healthy dietary pattern is associated with a lower lung cancer risk, and they provide more beneficial evidence for changing the diet pattern in the general population.

## 1. Introduction

Lung cancer is one of most common cancer forms worldwide, and it ranks as a top cause of cancer death [1]. Cigarette smoking is the primary risk factor for lung cancer, but other factors including advanced age, family history, air pollution, and radon and asbestos exposure may also increase the risk of lung cancer. The incidence of lung cancer is still rising in developing countries, e.g., China and India, which is mainly attributable to environmental risk factors [2,3]. With the arrival of target therapy and precision medicine, the outcome of lung cancer is still poor and disappointing [3,4,5]; therefore, prevention is the best strategy to lower its mortality.

It has gradually been recognized that diet and nutrients play an important role in cancer development and progress, and many dietary components are found to be associated with cancer risk [6]. With regard to lung cancer, high intakes of vegetables, fruits [7], fish [8] and soy intake [9] have been found to reduce lung cancer risk, while red meat and processed meat might increase its risk [10,11]. However, almost all the clinical intervention trials with isolated nutrients, such as vitamin A, vitamin E, vitamin C, folate [12], selenium and carotenoid [13] supplements, failed to demonstrate their protective effects against lung cancer; dietary supplementation with beta carotene alone is even linked to a higher lung cancer risk in smokers [14,15]. Since different foods are consumed in combinations, and they interact with each other in a complex way, a comprehensive diet pattern analysis can better reflect dietary habits and provides a constructive instrument to evaluate the overall effects of total diet on human health [16,17].

A healthy dietary pattern (also known as prudent pattern) is characterized by a high intake of vegetables, fruits, white meat, fish and whole-grain breads and a low intake of red meat, high fat and refined grains [18]. Previous studies have indicated that a healthy eating pattern might reduce the risk of cardiovascular disease and total mortality [19], and it has also been linked with a lower risk of several kinds of cancer [20,21,22]. Based on previous studies on the association between individual components and lung cancer risk, we proposed a hypothesis that a healthy dietary pattern might lower lung cancer risk. However, the results on the association between a healthy dietary pattern and lung cancer risk are inconsistent; Tsai *et al.* first reported the association with null results in the overall population [23], and then several following studies found different results showing that a healthy eating pattern might be associated with a lower lung cancer risk [24,25,26,27], while other studies found an insignificant inverse association between them [28,29,30]. For this reason, we searched relevant observational studies and conducted a systematic review with meta-analysis to investigate the effect of a healthy dietary pattern on lung cancer risk.

## 2. Materials and Methods 

### 2.1. Search Strategy

We conducted and reported our meta-analysis in accordance with the Meta-analysis of Observational Studies in Epidemiology (MOOSE) guidelines [31]. Related articles were searched in electronic databases, including PubMed and EMBASE, up to October 2015. The following key words including “lung neoplasm” or “lung cancer” or “lung carcinoma” or “non-small cell carcinoma” or “small cell carcinoma” or (lung$ or pulmon$ and (tumor$ or tumour$ or cancer$ or onco$ or carcinoma or neoplas$ or adenocarcinoma)) were used in combinations with “diet” or “nutrients” or “dietary pattern” or “dietary habits” or “food pattern” or “eating pattern” or “lifestyle”. No language restriction was applied (Table S1). We also reviewed the references of identified papers to find more relevant studies. 

### 2.2. Selection Criteria

Our interests were focused on the studies investigating the relationship between multiple food groups and lung cancer risk. There is still no accurate definition of a healthy dietary pattern, which is a relatively recent concept, in contrast with “western dietary (unhealthy) pattern” or “drinker dietary pattern”. The dietary patterns can be classified by a data-driven approach (*a posteriori* method), including factor analysis and cluster analysis, or an index-based approach using dietary guidelines (*a priori* method). In our study, healthy dietary patterns identified by two approaches were all included if they met the following criteria: (1) cohort or case-control design; (2) the outcome was lung cancer risk, including both incidence and mortality; (3) relative risk (or odds ratio or hazard ratio) with 95% confidence intervals could be obtained or calculated from data in the manuscript. When several publications were reported on the same population, the most recent and informative one was preferred in our meta-analysis.

### 2.3. Data Extraction and Quality Assessment

Two investigators (Yanlai Sun and Zhenxiang Li) independently reviewed all the studies and extracted the following information from the identified articles, including the first author’s name, publication year, duration, location, design, dietary assessment, dietary pattern identification method, RR with 95% CIs and adjusted variables. We also evaluated the quality of included studies by the Newcastle-Ottawa scale, which is widely used in assessing observational studies [32]. Briefly, each study was assigned a maximum of nine points, four for selection, two for comparability, and three for outcomes in cohort studies or exposures in case-control studies; a final score ≥7 was considered as high quality in our study. Discrepancies were discussed and resolved with the third investigator (Jianjun Han).

### 2.4. Statistical Methods

As described before, a healthy dietary pattern is defined by a combination of food groups, and can be varied a bit among different studies, which might also be labeled as “healthy” [23,26,27,29] or “prudent” [30] or “salad vegetables” [28] or “vitamins and fiber” [25] or “high RFS (recommended foods score) dietary pattern” [24]. In original studies, the results were always reported in terms of tertiles, quartiles or quintiles of dietary scores based on factor analysis or dietary guidelines. The RRs with their 95% CIs comparing the highest with the lowest categories of the healthy patterns were extracted from the original studies. When separated RRs were reported stratified by sex, overall RRs were combined by a fixed-effects model. We tested heterogeneity across studies using the Q and *I*^2^ statistics; for the Q statistic, if *p* < 0.1, significant heterogeneity was considered to exist, and in this case the random-effects model was used to pool the original RRs. Otherwise, the fixed-effects model was employed. We tried to conduct subgroup analyses stratified by confounding factors to test the stability of the results, and also performed a sensitivity analysis in which the individual study was removed to examine the influence of a specific study on the overall results. Publication bias was evaluated by the use of the funnel plot and Egger’s test, with *p* < 0.1 representing a significant publication bias. All statistical analyses were conducted with STATA 12.0 software (Stata Corporation, College Station, TX, USA). 

## 3. Results

### 3.1. Literature Search and Study Characteristics

**Figure 1:**
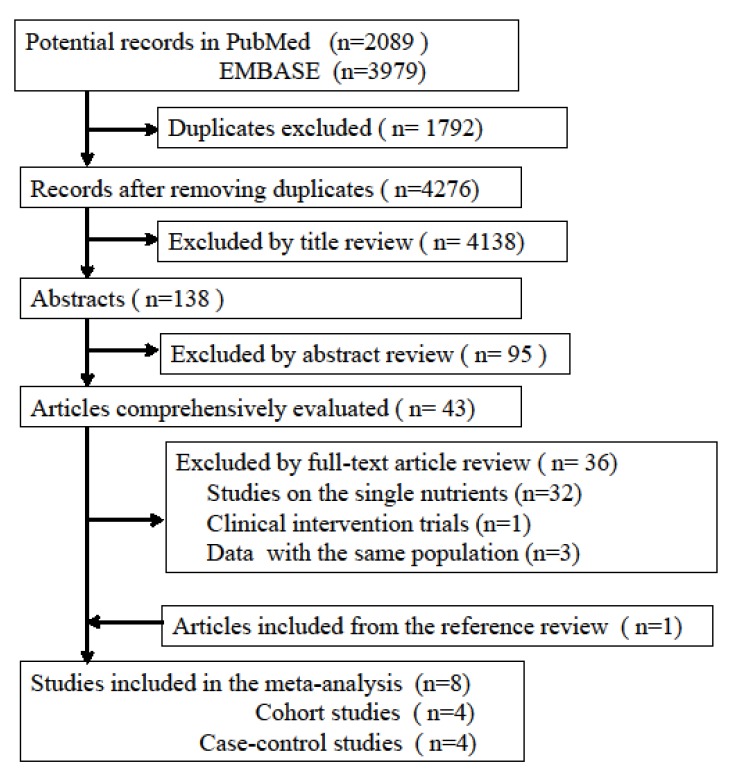
Flow chart of literature search.

Initially, 4276 records were found in the electronic databases; after a process of careful checking and review, a total of eight studies, involving 13,548 lung cancer cases and 108,748 participants, were finally included in our meta-analysis. They included four case-control studies and four cohort studies. The literature search flow is shown in Figure 1, and the characteristics of the original studies are listed in Table 1. Among all the studies, four were from the USA, two from Uruguay, two from Europe, and no studies were from Asia. Three studies [11,33,34] were excluded in our study because of replicate reporting or no data on healthy patterns; particularly, one study by De Stefani *et al.* [34] only checked the relationship between lung adenocarcinoma risk and dietary pattern, and the cases might be from the same population as used in previous studies [29,30].

Overall, the quality scores of the original studies ranged from 6 to 9, with an average of 7.38 points. Eight of nine studies were judged as high quality, and no low quality studies were found. All the studies employed food frequency questionnaires (FFQs) in dietary assessment, and five of them were validated [23,24,25,27,28]. In most studies, healthy patterns were classified by *a posteriori* data-driven approaches, such as principal component factor analysis [25,26,28,29,30] or cluster analysis [23], and the other two studies used *a priori* methods based on the HEI (healthy eating index)-2010 Score [27] or the Recommended Foods Score (RFS) [24]. The results in all the studies were adjusted for the most common confounders, including smoking status, age, gender and total energy intake.

### 3.2. Meta-Analysis

**Figure 2:**
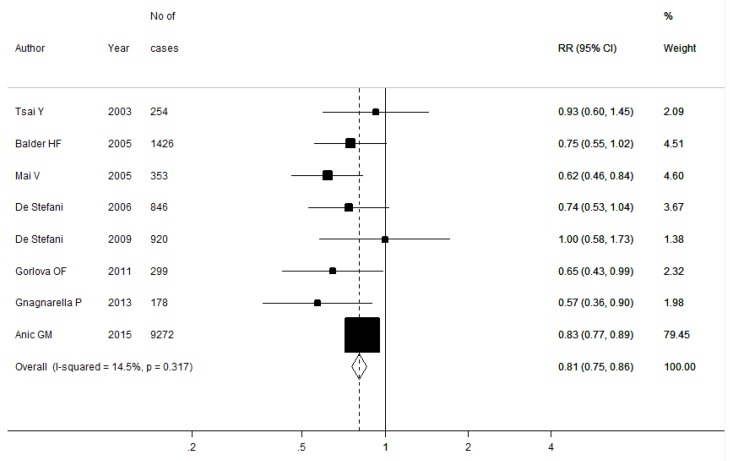
Forest plot of heathy dietary pattern and lung cancer risk.

No heterogeneity was detected among all the included studies (*I*^2^ = 14.5%, Q = 8.18, *P =* 0.32); thus, the fixed-effects model was used. A summary RR of 0.81 (95% CI: 0.75–0.86) was yielded after combining all the results (Figure 2). The RRs were 0.79 (95% CI: 0.64–0.97) for case-control studies and 0.73 (95% CI: 0.61–0.87) for cohort studies, respectively.

### 3.3. Subgroup Analyses

**Figure 3:**
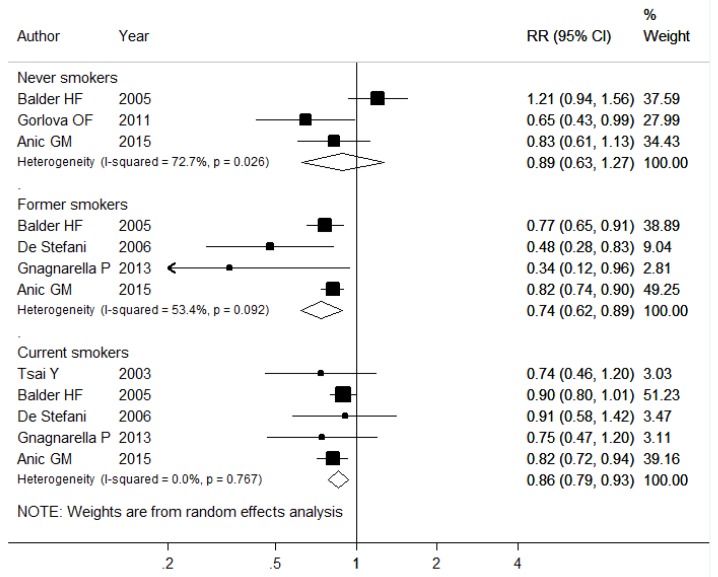
Forest plot of heathy dietary pattern and lung cancer risk, stratified by smoking status.

In a subgroup analysis stratified by smoking status, the inverse association was strengthened in former smokers (RR: 0.74, 95% CI: 0.62–0.89), while it disappeared in never smokers (RR: 0.89, 95% CI: 0.63–1.27); possibly due to the small number of the sample size, the pooled RR for current smokers was 0.86 (95% CI: 0.79–0.93) (Figure 3). After stratifying by sex, the RRs for males and females were 0.85 (95% CI: 0.63–1.15) and 0.63 (95% CI: 0.48–0.82), respectively (Figure S1). Besides, when we only analyzed the six studies using the *a posteriori* method, the combined RR was 0.75 (95% CI: 0.64–0.88), and the pooled RR for studies using validated FFQs was 0.76 (95% CI: 0.65–0.88), indicating the robustness of the results (Figure S2).

Not enough data were obtained on the histological subtypes of lung cancer; however, the studies by Gnagnarella *et al.* [25] and De Stefani *et al.* [34] in 2011 both reported that a healthy pattern reduced the lung adenocarcinoma risk significantly, and the results were quite similar, with RRs of 0.53 (0.31–0.91) and 0.54 (0.32–0.92), respectively. 

### 3.4. Sensitivity Analysis and Publication Bias

**Figure 4:**
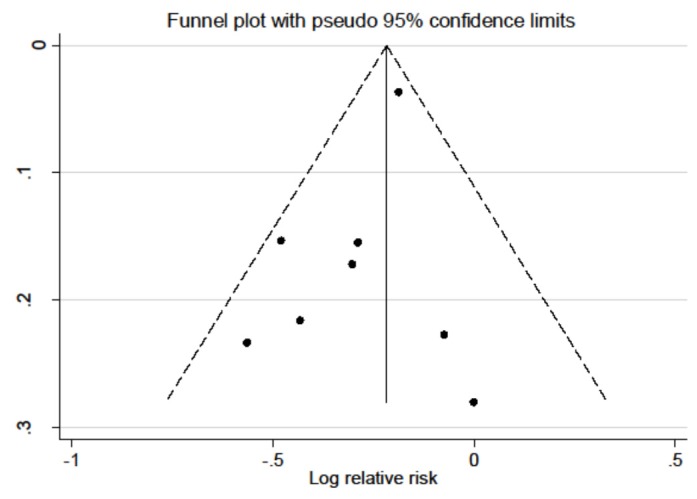
Funnel plot for all studies included in the meta-analysis.

When a sensitivity analysis was carried out by removing individual studies each time, the recalculated RRs using a fixed-effects model remained stable; however, after omitting the study by Anic [27], the pooled RR was 0.72 (0.62–0.83) with no heterogeneity found (*I*^2^ = 0.00%, Q = 4.9, *p =* 0.56) in this scenario (Figure S3). No implication of publication bias was detected by the Egger’s test (*p* = 0.18), although the funnel plot seemed slightly unsymmetrical (Figure 4), indicating that there was no significant publication bias in our meta-analysis.

## 4. Discussion

As far as we know, this is the first meta-analysis and systematic review on the association between dietary patterns and lung cancer risk. Our analysis suggests that a healthy dietary pattern may reduce lung cancer risk, with little heterogeneity across studies. The results are well established in subgroup analyses stratified by gender, smoking and dietary pattern identification method, with the exceptions of the subgroups of non-smokers and males, mainly due to a small amount of studies included. 

Previous studies have found that a healthy eating pattern is helpful in reducing the risk of several types of cancer, including colorectal cancer [35], breast cancer [21], esophageal squamous cell carcinoma [22] and gastric cancer [20,36]. Since dietary pattern analysis can better reveal one’s dietary habit and the interaction of foods, the conclusion was more convincing than the results on isolated nutrients, thus providing more evidence on the benefits of a healthy eating pattern in lung cancer prevention. In fact, the vital components of a healthy eating pattern, including vegetables [37], fruits [7], fish [8], white meat [10], and soy foods [9], have all been reported to be associated with a lower lung cancer risk. The results of our meta-analysis reveal that the healthy foods combined together can also fight against lung cancer, probably more efficiently than any of the separate foods can alone. The mechanism here is undoubtedly related to anti-tumorigenic agents contained in the individual components of a healthy diet, including antioxidants, polyphenols, fiber and minerals; moreover, their interrelations might synergistically enhance their individual protective effects as a whole. For example, recent studies also showed that dietary pattern could influence gut microbiome [38,39], which was also associated with several gastrointestinal cancers [40]. However, the overall effects of different dietary patterns on human health still need more investigation.

Meta-analysis is a statistical method of combining the results of original studies, making the conclusion more convincing. The advantages of our study included a large sample size (more than 10 thousand lung cancer cases and 100 thousand participants), and a broad time span from 1986 to 2010. Additionally, subgroup analyses by gender, smoking status and dietary pattern analysis method were also performed to minimize the effects of confounding factors. 

However, some shortcomings should be also recognized in our study. Firstly, different methods for dietary assessment and pattern analyses were employed in the original studies; particularly, the FFQs used in several studies [26,29,30] were unvalidated, and the results of dietary pattern analyses based on factor analysis are always distinctive to each study, making them uncomparable to some extent. All these factors contributed to heterogeneity across studies; however, when tested by the Q test, the heterogeneity was not significant and mainly came from the study by Anic *et al.* [27], and when the study was removed, the results were not highly influenced. 

Secondly, recall and selection bias were inevitable in the observational studies, especially for hospital-based case-control studies. Inadequate adjustment for potential confounders including social-economic status, physical activity, and total energy intake also influenced the reliability of the results, because healthy eating might be a sign of other aspects of healthy lifestyles, including more exercise, positive attitude, better medical service, and less frequent smoking and drinking. In addition, given the limited number of studies included and insufficient data, subgroup analyses by histological types and ethnicity were impossible to carry out. Especially there is still no data from Asia, including China, Japan or India. Notably, compared to the Western dietary pattern, the Asian food pattern generally may fall under “healthy diet”. Thus, more studies to explore the relationship between Asian diet and lung cancer risk are required.

Lastly, since our literature was limited to publications in English, and studies with null results tend to not be published, this might cause incomplete articles to be included in the meta-analysis; however, no significant publication bias was found by Egger’s test, suggesting the influence was acceptable. 

In contrast with healthy pattern, we also attempted to combine all the results on the association between Western dietary pattern and lung cancer risk in five published articles [11,28,29,30,33]. As was expected, the pooled RR was 1.77 (95% CI: 1.37–2.28), with a substantial heterogeneity (*I*^2^ = 59.8%, *p =* 0.04), indicating that bad eating habits might increase lung cancer risk significantly. In addition, the Mediterranean diet pattern, which is considered a special type of healthy eating pattern, has also been found to reduce lung cancer risk in two studies [11,27]. Thus, although smoking cessation is still the most effective approach to lowering lung cancer incidence, healthy eating and lifestyle are also necessary for the prevention of lung cancer, especially for former and current smokers.

## 5. Conclusions

In summary, findings from the meta-analysis of observational studies suggest that a healthy dietary pattern is associated with lower lung cancer risk and provides more beneficial evidence for changing the diet pattern in the general population. Given the limited number studies included, more prospective studies with strict control of confounders or interventional trials, especially in Asia, are needed to confirm this association.

## Figures and Tables

**Table 1:** Characteristics of studies included in the meta-analysis on the association between healthy dietary pattern and lung cancer risk.

Author Year	Duration	Design	Location	Study Population	Sex	No. of Case	Cohort Size/Control	Dietary Assessment	Dietary Patterns Identification Method	Dietary Pattern Characteristics	Dietary Patterns Identified	RR (Highest to Lowest)	Adjusted Variables	Quality
Tsai Y., 2003 [24]	1995–1996	Case-control	USA	Patients seen at Fox Chase Cancer Center Network	Both	254	184	FFQ (food frequency questionnaire)-61 items, validated, self-reported	Posteriori, cluster analysis	A relatively greater intake of carbohydrates and dietary fiber and a lower intake of animal fat and protein.	Healthy pattern	0.93 (0.59–1.44)	Gender, age, and smoking	7
Balder H.F., 2005 [30]	1986–1995	Cohort	Europe	The Netherlands Cohort Study on Diet and Cancer	Male	1426	58,279	FFQ-150 items, validated, interviewed	Posteriori, principal components analysis	High factor loadings on several vegetable items, several fruit items, pasta, rice, poultry, fish, and oil	Salad vegetables	0.75 (0.55–1.01)	All other dietary patterns and age at baseline, total energy intake, current cigarette smoker, number of cigarettes smoked per day, years of smoking cigarettes, higher vocational or university education, family history of lung cancer, physical activity	9
Mai V., 2005 [32]	1987–1998	Cohort	USA	The Breast Cancer Detection Demonstration Project cohort	Female	353	42,254	FFQ-62 items, validated, mailed	Priori	Increasing consumption of fruits, vegetables, whole grains, lean meats or meat alternatives, and low-fat dairy.	High RFS (the Recommended Foods Score) dietary pattern	0.62 (0.46–0.84)	Energy intake, smoking, NSAID (nonsteroidal anti-inflammatory drug) use, and BMI (body mass index), smoking duration and cigarettes/day	8
De Stefani, 2006 [28]	1995–2001	Case-control	Uruguay	Patients from four major hospitals located in Montevideo, Uruguay	Male	846	846	FFQ-64 items, unvalidated, interviewed	Posteriori, principal component analysis	High correlations of white meat, fresh vegetables, cooked vegetables, citrus fruits and non-citrus fruits	Healthy pattern	0.74 (0.53–1.04)	Age, residence, urban/rural status, education, family history of lung cancer among first-degree relatives, body mass index, cigarettes per day, years since quit and total energy intake.	7
De Stefani, 2009 [29]	1996–2004	Case-control	Uruguay	Patients from four major hospitals in Montevideo, Uruguay	Both	920	2532	FFQ-64 items, unvalidated, interviewed	Posteriori, principal component analysis	High positive loadings for poultry, fish, fresh vegetables, and total fruits.	Prudent pattern	1.00 (0.58–1.74)	Age, residence, urban/rural status, education, body mass index, smoking status, years since stopping, number of cigarettes/day, among current smokers, total energy intake and all the dietary patterns	7
Gorlova O.F., 2011 [27]	1995–2008	Case-control	USA	Patients in MD Anderson Cancer Center	Both	299	317	FFQ-214 items, unvalidated, interviewed	Posteriori, a principal component-based factor analysis	High intake vegetables, fruits, and low fat products.	Healthy pattern	0.65 (0.42–0.98)	Age, gender, caloric intake, and education	6
Gnagnarella P., 2013 [31]	2004–2010	Cohort	Italy	The COSMOS screening study, current smokers or former smokers	Both	178	4336	FFQ-188 items, validated, self-administered	Posteriori, principal component analysis	High intake of vegetables, fruits, nuts, cereals, legumes and fish; low consumption in red and processed meat	Vitamins and fiber	0.57 (0.36–0.90)	Age, sex, smoking history, asbestos exposure and total energy	7
Anic G.M., 2015 [26]	1995–2006	Cohort	USA	The NIH–AARP Diet and Health Study	Both	9272	460 770	FFQ-124 items, validated, mailed	Apriori, HEI(healthy eating index)-2010 score	High intake of total vegetables, greens and beans, total fruits, whole fruits, seafood, whole grains and low-fat dairy	Healthy eating pattern	0.83 (0.77–0.89)	Age, sex, race, education, body mass index, physical activity, total energy, smoking status, cigarettes per day, time since quitting smoking and regular use of cigars/pipes	8

Abbreviations: FFQ: food frequency questionnaire; RFS, the Recommended Foods Score; HEI, healthy eating index; NSAID: nonsteroidal anti-inflammatory drug; BMI: body mass index.

## Supplementary Materials

The following are available online at http://www.mdpi.com/2072-6643/8/3/134/s1, Table S1. Search strategy in electronic databases. Figure S1. Forest plot of healthy dietary pattern and lung cancer risk, stratified by gender. Figure S2. Forest plot describing subgroup analysis of the studies used in factor analysis on the association between healthy dietary pattern and lung cancer risk. Figure S3. Sensitivity analysis for the association between healthy dietary pattern and lung cancer risk.
