# Supplementary material for: A Healthy Dietary Pattern Reduces Lung Cancer Risk: A Systematic Review and Meta-Analysis
Source: Nutrients. 2016 Mar 4;8(3):134. doi: 10.3390/nu8030134 (PMC4808863; doi:10.3390/nu8030134)
Supplement: Supplementary file 1 [file nutrients-08-00134-s001.docx]

**Supplementary Materials: A Healthy** **Dietary Pattern Reduces** **Lung Cancer Risk: A Systematic Review and Meta-Analysis**

Yanlai Sun, Zhenxiang Li, Jianning Li, Zengjun Li and Jianjun Han

**Table S1.** Search strategy in electronic databases.

| 1. “lung cancer” |
| --- |
| 2. “lung carcinoma” |
| 3. “lung neoplasm” |
| 4. “non-small cell carcinoma” or “NSCLC” or “small cell carcinoma” or “SCLC” |
| 5. lung$ OR pulmon$ AND (tumor$:ab,ti OR tumour$:ab,ti OR cancer$:ab,ti OR onco$:ab,ti OR carcinoma:ab,ti OR neoplas$:ab,ti OR adenocarcinoma:ab,ti) |
| 6. 1 OR 2 OR 3 OR 4 OR 5 |
| 7. diet OR “dietary pattern” OR “dietary habits” |
| 8. nutrients or nutrition |
| 9. “food pattern” or “eating pattern” |
| 10. lifestyle |
| 11. 7 OR 8 OR 9 OR 10 |
| 12. 6 AND 14 |

“$”: for any character, ab: abstract; ti; title.


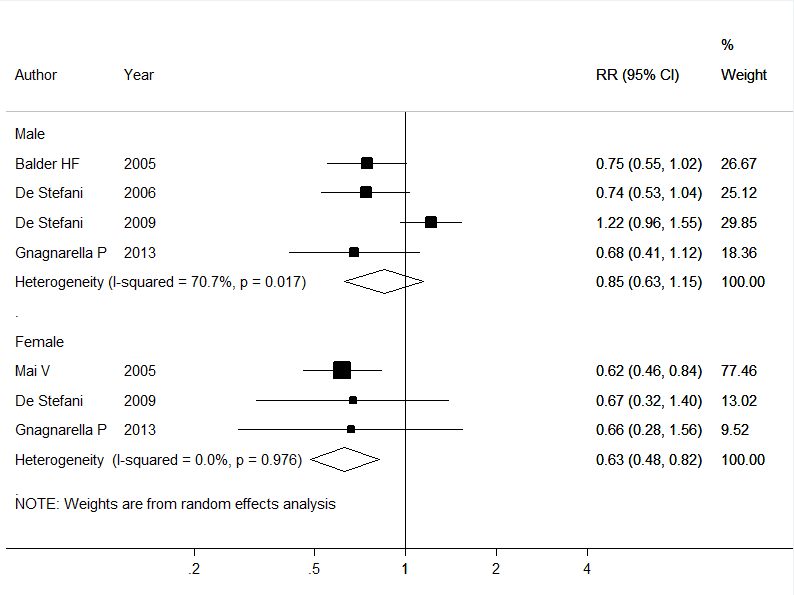


**Figure S1.** Forest plot of healthy dietary pattern and lung cancer risk, stratified by gender.


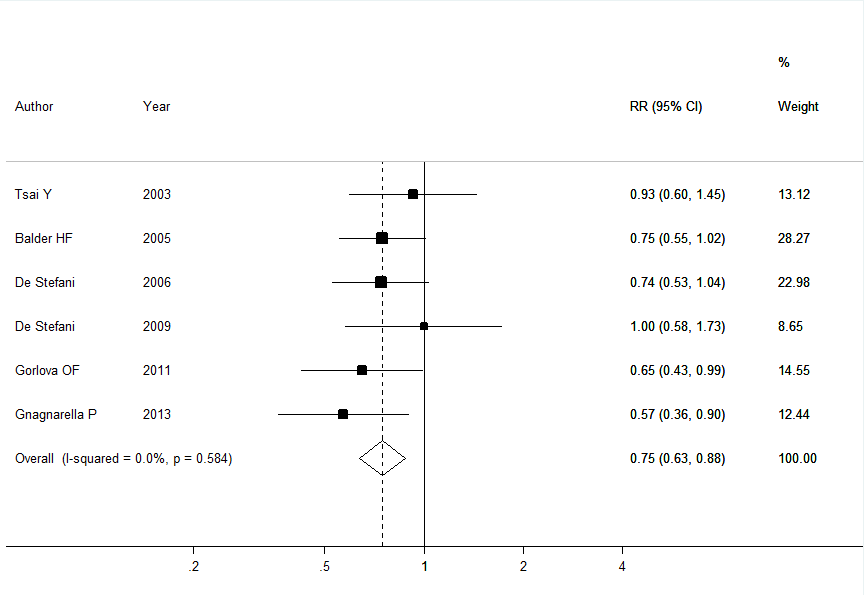


**Figure S2.** Forest plot describing subgroup analysis of the studies used factor analysis on the association between healthy dietary and lung cancer risk.


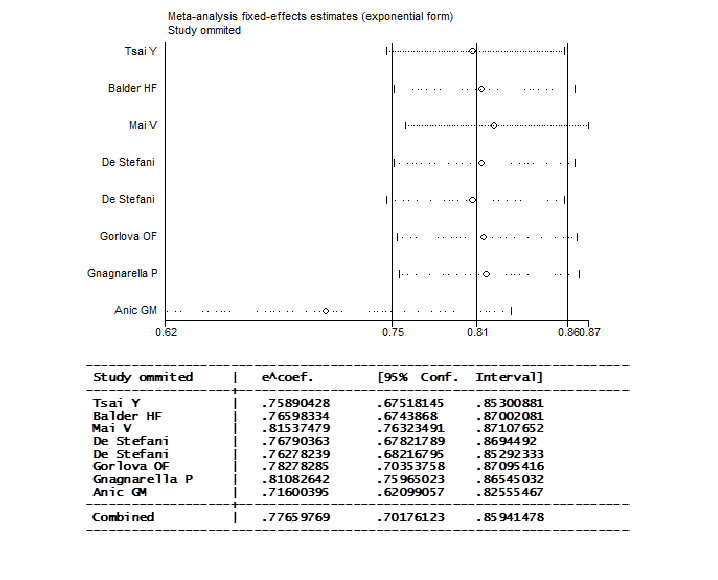


**Figure S3.** Sensitivity analysis for the association between healthy dietary pattern and lung cancer risk.
